# Autistic Traits and Somatic Symptom Disorders: What Is the Link?

**DOI:** 10.3390/brainsci14030274

**Published:** 2024-03-14

**Authors:** Barbara Carpita, Benedetta Nardi, Valeria Tognini, Francesca Poli, Giulia Amatori, Ivan Mirko Cremone, Stefano Pini, Liliana Dell’Osso

**Affiliations:** Department of Clinical and Experimental Medicine, University of Pisa, 67 Via Roma, 56126 Pisa, Italy; barbara.carpita1986@gmail.com (B.C.); valeria_tog@hotmail.com (V.T.); fra.poli13@gmail.com (F.P.); ivan.cremone@gmail.com (I.M.C.); stefano.pini@unipi.it (S.P.); liliana.dellosso@gmail.com (L.D.)

**Keywords:** autism spectrum disorder, autism, autistic traits, somatic symptom disorder, somatic symptoms, somatic symptom and related disorders

## Abstract

Alterations in sensory processing, a key component of autism spectrum disorder (ASD), have recently attracted increasing attention as they result in peculiar responses to sensory stimuli, possibly representing a risk factor for the development of somatic symptom disorder (SSD). Contextually, other features also associated with ASD, such as alexithymia, camouflaging and altered verbal, and non-verbal communication, have been suggested to represent risk factors for the occurrence and worsening of somatic symptomatology. The aim of this work was to review the available literature about the association between SSD and the autism spectrum. The results highlighted not only a higher prevalence of autistic features in patients suffering from SSD and a higher prevalence of reported somatic symptomatology in subjects with ASD but also how ASD subjects with co-occurrent somatic symptoms exhibit more severe autism-linked symptomatology. From the paper reviewed also emerged many shared features between the two conditions, such as alexithymia, altered sensitivity to sensory stimuli, cognitive inflexibility, intolerance of uncertainty, and an increased risk of experiencing stressful life events, which may provide an explanation for the correlation reported. Even though studies on the topic are still scant, the evidence reported suggests the importance of further assessing the correlation between the two disorders.

## 1. Introduction

Autism spectrum disorder (ASD) is a neurodevelopmental condition characterized by repetitive and restricted interests, behavioral patterns, and persistent difficulty in social interactions and communication [1]. An alteration in sensory processing, which happens when the capacity to behave in response to sensory information such as sound, touch, body movement, sight, taste, and smell is impaired, is a key component of ASD that has recently attracted increasing attention. This change may eventually result in peculiar responses to sensory stimuli, which would interfere with the daily activities of ASD people [2]. The autism spectrum, aside from its core symptoms, is also frequently associated with other features, such as alexithymia and anxiety [3,4]. Despite ASD mainly being studied in children, studies on adults are equally important, particularly because the autism spectrum is hypothesized to be a risk factor for the development of other mental conditions [5,6]. In this framework, a topic of utmost clinical interest is the possible presence of undetected ASD among adult patients seeking treatment, highlighting the importance of investigating autistic symptoms in both clinical samples and the general population [7]. Research on ASD in recent years has emphasized the need to examine not only full-blown conditions but also milder, sub-clinical manifestations of the autism spectrum, which are known under the name of the “broad autism phenotype” due to their first investigation being among first-degree relatives of ASD probands and which appear to be distributed along a continuum from the general to the clinical population [5,6,8,9].

According to the American Psychiatric Association [1], somatic symptom disorder (SSD) is characterized by one or more somatic symptoms that are bothersome or significantly interfere with daily life, as well as excessive thoughts, feelings, or actions associated with those symptoms. Although the length and severity of SSD vary depending on the patient, persistent SSD can impair everyday functioning and result in more frequent medical examinations, school absences, and emotional anguish for both the kid and the parents [10,11]. A history of psychiatric disorders in the family and during childhood [12], stressful life events, and pain hypersensitivity [13] are risk factors for SSD, as are an older age, female gender [14], and the style of coping with stress, including alexithymia [15]. Noticeably, some features associated with the autism spectrum, such as alexithymia [16], altered sensitivity to pain [17], and increased risk of experiencing stressful life events, may also be considered, as listed above, risk factors for SSD. Due to their social impairment and decreased socioemotional reciprocity, people with ASD are more likely to experience socially unpleasant or even traumatic situations, bullying, and rejection, which increase their risk of developing trauma- and stress-related illnesses. Additionally, it has been noted that ASD people typically show difficulties in identifying, processing, and externalizing traumatic experiences, with a reduced ability to cope with stressful situations [5,6]. This characteristic may make subjects on the autism spectrum more vulnerable to experiencing stress-related symptoms in response to less severe life events, but it also increases the likelihood that PTSD symptoms in this population would go unreported and undiagnosed [5,6]. According to the scientific literature, under-reported traumatic experiences have been linked to somatic symptom and related disorders (SSRDs), which, as reported above, are more common in women. Noticeably, increasing research is stressing the presence of gender-specific presentations of the autism spectrum, with female-specific characteristics being often under-recognized [5,18,19,20,21,22]. Females on the autism spectrum often show a reduced impairment in social interaction, partly due to their greater ability to mask their symptoms using social camouflaging strategies, but with higher levels of social anxiety as a consequence [18,19,20,21,22,23]. Moreover, they seem to show restricted interests more oriented towards animals or people, such as spending time with animals, enjoying fiction, and focusing on food and diet [18,19,20,21,22]. Intriguingly, patients with ASD, particularly females, often show a higher prevalence of somatic symptoms, possibly as a result of the challenges with mentalizing and expressing psychological discomfort [18]. Despite that, to date, limited research focused on the relationship between autism spectrum disorder and SSRDs has been carried out. An eventual association between these conditions may suggest the need to investigate the relationship between SSRDs and female phenotypes of the autism spectrum [18,19,20,21,22,24]. In addition, considering the common ground of alexithymia between SSRDs and ASD, the dramatic and sometimes artificial communication style that is reported among patients with SSRDs could be linked with a failing attempt to utilize social camouflaging techniques to compensate for their impaired communication and socioemotional reciprocity. Notably, BPD, another disorder associated with the female gender, autistic features, and a history of trauma, is known to frequently co-occur with both FED and SSRDs [22,24,25,26]. In this theoretical framework, the aim of this work was to review the available literature about the association between SSRDs and autism spectrum disorder.

## 2. Methods

Using the electronic database PubMed, a literature search was carried out between 1 October 2023 and 20 December 2023. To find all potentially eligible records, the following search terms were used, without any filters, restrictions, or limits: “alexithymia AND somatoform disorders”, “autism spectrum disorders AND somatoform disorders”, and “alexithymia AND autism spectrum disorders”. Two independent psychiatrists conducted the article search, and disagreements were resolved by discussion. The level of agreement between the two reviewers was good.

### 2.1. Inclusion Criteria

- Based on the type of article, original studies, editorials, and case report were accepted;
- Studies conducted on humans;
- Full text available in English.

### 2.2. Exclusion Criteria

- Reviews or meta-analyses;
- Studies conducted on animals;
- Articles not available in English.

## 3. Results

### 3.1. Autistic Traits in SSRDs

Hatta et al. [27] were the first authors to quantitatively compare autistic symptoms in children with SSD with classmates who were typically developing. Before that, the literature was limited to case reports stressing the presence of higher levels of autistic traits among children with somatization [28,29,30]. The authors compared the presence of autistic traits by using the Autism Spectrum Quotient (AQ), the children’s version. The final study sample included 26 control participants (F = 12; M = 14; mean age: 12.7 ± 1.9 years) and 28 children with SSD (F = 15; M = 13; mean age: 13.4 ± 2.0 years). No significant differences were found for age and gender between the groups. In comparison to the control group, the SSD group had a considerably greater prevalence of a family psychiatric history. While the SSD group scored higher on the AQ than the controls, the difference was not statistically significant for the total score but only for the AQ “Attention Switching” domain. Moreover, 42.9% of the individuals with SSD reported an AQ score above the average (1 SD above the mean). Noticeably, higher scores on the AQ “Attention Switching” domain were also associated with reduced health-related quality of life, especially in the area of family and friends, as measured by the KINDL.

Nisticò et al. [31] assessed 45 neurotypical adults (NAs), 30 people with ASDs who did not have intellectual disabilities, and 21 patients with functional neurological disorders (FNDs) with the Ritvo Autism Asperger Diagnostic Scale—Revised (RAADS-R), the AQ, and a questionnaire measuring functional neurological symptoms (FNSs). The participants with ASDs additionally filled out the Sensory Perception Quotient–Short Form (SPQ-SF35), which measures sensitivity to sensory stimuli. A significant difference was observed in the prevalence of FNSs, with 86.7% of the participants with ASDs reporting at least one, compared to 35.6% of the NA participants (*p* < 0.001). Moreover, it was discovered that while the individuals with ASDs displayed a higher number of FNSs than the NAs, the individuals with FNDs did not exhibit more autistic traits than the NAs; this rate was linked to increased sensory sensitivity, particularly in the touch domain.

While several studies have suggested in SSRDs a possible increased presence of alexithymia, with the latter being linked, similarly to ASD, to deficits in empathy and impaired reading of facial expressions [32,33,34,35], some authors also hypothesized that the difficulties in reading, understanding, and communicating emotions may result in a more frequent somatic expression of mental suffering [34]. In a 2023 study, Cole et al. [36] aimed to evaluate the presence and correlates of autistic traits in a group of patients with functional neurological disorder (FND), considering a possible mediating role of alexithymia between autistic traits and somatic symptoms. They investigated 91 patients (F = 69, M = 22 males, mean age = 43.42 ± 13.39 years) with FND, grouping them on the basis of an AQ-10 score <6 or ≥6. All the subjects were assessed with psychometric questionnaires, including the Somatic Symptom Questionnaire (PHQ-15), the Patient Health Questionnaire (PHQ9), the Adult ADHD Self-Report Scale (ASRS), the Adult Dyslexia Checklist, the Toronto Alexithymia Scale (TASS-20), the Generalized Anxiety Disorder-7 (GAD-7), the Social Phobia Inventory, and the Work and Social Adjustment Scale (WSAS). The patients with a high AQ score (40% of the sample) reported significantly higher scores for dyslexia, ADHD, generalized anxiety disorder, depression, social phobia, and alexithymia. When splitting the sample depending on the alexithymia score, the subjects with a higher alexithymia score showed higher levels of dyslexia, generalized anxiety disorder, depression, somatic symptom severity, social phobia, and autistic traits. The levels of severity of somatic symptoms, generalized anxiety, sadness, social phobia, and dyslexia were all considerably higher in the alexithymia-positive patients. The association between autistic characteristics and PHQ9 depression scores was discovered to be mediated by the alexithymia score. However, the AQ-10 score showed a significant direct effect on the presence of somatic symptoms, as measured by the PHQ15, without a significant indirect effect on the alexithymia score.

Regarding the specific issue of non-epileptic seizures, a 2016 case study by Miyawaki et al. [37] from Japan described the case of a 10-year-old girl with undiagnosed ASD. While receiving therapy for benign childhood epilepsy with centrotemporal spikes, she developed psychogenic non-epileptic seizures (PNESs). PNESs seem to be common in children with epilepsy, often occurring among subjects with underlying psychological distress. The patient displayed social communication deficits, hypersensitivity to sound, and interpersonal conflicts. Following an intervention focused on reducing her distress due to auditory hypersensitivity and impaired social communication—both traits are associated with ASD—her PNES symptoms improved quickly. The authors highlighted that, among children with undiagnosed ASD, PNESs may be the outcome of psychological distress.

Moreover, a further study by McWilliams et al. [38] examined the medical records of 59 patients under the age of eighteen who were referred to a pediatric mental health service specialist for the evaluation and management of non-epileptic seizures between 2012 and 2016. The findings indicated that 10/59 (16.9%) of those with non-epileptic seizures also had ASD and that half of them were not diagnosed with ASD before the referral.

A summary of the described studies is shown in Table 1.

### 3.2. SSRDs in ASD

In a study conducted in Sweden in 2019 by Asztely et al. [39], a group of women with ASD were examined for the presence of chronic pain and its relationship to health-related quality of life. The sample consisted of a group of 77 women aged between 19 and 37 years using standardized tests of pain perception and quality of life, like the Short-Form Health Survey (SF-36). The results showed that 76% of the women examined reported chronic pain and, in general, a low quality of life, which was lower among those with chronic pain.

Zdankiewicz-Cigaa et al. led a study in 2021 with the aim of assessing the relationship between somatoform symptoms and interoceptive sensibility in ASD subjects, hypothesizing that the higher interoceptive sensibility in people with ASD may be associated with greater alexithymia in the emotions’ identification and verbalization dimensions [40]. The authors enrolled a total of 205 subjects (F = 157; M = 48; mean age: 34.91 ± 8.44 years); the sample included a clinical group (N = 79) with Asperger’s syndrome and an AQ score of at least 32 and a control group (N = 126) that had never received a diagnosis on the autism spectrum. The level of alexithymia was investigated using the Toronto Alexithymia Scale (TAS-20), the autonomic reactivity and bodily awareness through the bodily Perception Questionnaire–Short Form (BPQ-SF), the emotional regulation through the Difficulties in Emotion Regulation Scale (DERS), and the Somatoform Dissociation Questionnaire (SDQ-20) for measuring somatoform dissociative symptoms. The clinical group reported significantly higher scores on the AQ, TAS-20, BPQ-SF autonomic reactivity subscale, SDQ-20, and DERS. According to a moderation analysis performed by the authors, the higher the score on the autonomic reactivity scale, the higher the score on the scale measuring somatoform symptoms, with a significantly higher effect strength in the clinical group. Performing further analysis, the authors also reported a significant indirect effect of autonomic reactivity on somatoform symptoms with alexithymia as a mediator [40].

In 2022, Larkin et al. [41] conducted an online study where somatic symptom rates, measured by the Patient Health Questionnaire-15, were compared among 202 older adolescents and adults (F = 146; M = 48; other = 8; mean age = 31.33 ± 12.90) with diagnosed ASD (N = 51), self-suspected ASD (N = 32), and without ASD (N = 119) while accounting for medical conditions. The authors investigated possible risk factors for somatic symptoms such as alexithymia, interoceptive sensibility, and intolerance of uncertainty with the General Alexithymia Factor Score, Body Awareness Questionnaire, and Intolerance of Uncertainty Scale, respectively. The study’s findings revealed that the people with ASD, controlling for physical and mental health issues, were more likely to show somatic symptoms than the non-ASD participants. The participants with self-suspected ASD reported intermediate levels of symptoms without showing significant differences with respect to the ASD and non-ASD groups. The non-ASD subjects showed significantly lower levels of alexithymia and interoception than both the ASD and self-suspected ASD groups, while the self-suspected ASD group showed significantly lower levels of interoception than the ASD group. Moreover, besides the female gender, physical and mental health issues, alexithymia, and intolerance of uncertainty also predicted somatic symptoms. No significant predictive effect was found, however, for interoception or the presence of an ASD diagnosis [41].

In a further study from 2022, Williams et al. assessed, in a cohort of transition-aged autistic young people from the USA, the prevalence, significance, and clinical correlations of fourteen commonly reported somatic symptoms [42]. The entire sample consisted of 290 subjects (F = 177; M = 113), aged between 18 and 26 years. A modified version of the Patient Health Questionnaire-15 was used in order to evaluate the presence and impact of somatic symptoms. Autistic traits were measured by the Social Responsiveness Scale, II edition (SRS-II), anxiety was measured by Generalized Anxiety Disorder-7 (GAD-7), depression symptoms were measured by the Beck Depression Inventory II (BDI-II), and quality of life was measured by the World Health Organization Quality of Life-4 (WHOQOL-4). The results showed that, in comparison to previous reports obtained for the general population, the somatic symptom burden was significantly higher in young adults with ASD. The three symptoms most frequently reported were exhaustion (72.8%), sleep issues (69.0%), and menstruation issues (61.4%, only females). Moreover, 53.9% of the females and 18.75% of the males reported moderate-to-severe symptom levels. Females were two to four times more likely than males to report a symptom. In addition, greater levels of anxiety, depression, and autistic features were linked to both individual symptoms and the total symptom burden, as well as to a lower quality of life [42].

A summary of the described studies is shown in Table 2.

## 4. Discussion

The present study collects the available evidence of an association between SSD and autism, which, in recent years, has been reported both in adult and child populations.

To date, many studies have described both a higher prevalence of autistic features in patients suffering from SSD [27] and a higher prevalence of reported somatic symptomatology in subjects with ASD [31,37,38,39,41,42]. Although the way in which autistic traits and somatoform disorders influence each other is still not entirely clear, it is widely recognized that ASD subjects with co-occurrent somatic symptoms exhibit more severe autism-linked symptomatology, like anxiety and externalizing behavior issues, and an overall lower quality of life [43,44,45]. Noticeably, some features associated with ASD, such as alexithymia [16], altered sensitivity to sensory stimuli [17], and an increased risk of experiencing stressful life events, may be considered risk factors for SSD. In this context, many studies have focused on exploring the reasons behind the frequent co-occurrence of SSD and autistic traits, providing diverse etiopathogenic hypotheses.

For instance, patients suffering from SSD were reported to show significantly higher attention switching difficulties compared to healthy controls, which may be explained by cognitive inflexibility that may ultimately lead to fixation on bodily sensations, heightening the intensity of somatic symptoms [27]. Secondly, it has been hypothesized that alterations in sensitivity to sensory stimuli can possibly influence the perception of somatic symptoms [46]. Indeed, autistic people’s interoceptive systems are known to function differently, leading them to exhibit both an increased sensitivity to physiological sensations and a decreased capacity to accurately detect bodily signals [47]. Based on these premises, the higher interoceptive sensibility of people with ASD may contribute to the greater prevalence of somatic symptoms in said population [40]. Lastly, a growing body of evidence is highlighting alexithymia and intolerance of uncertainty as predictors not only of the severity of autistic symptomatology [3,48,49] but also of the presence of somatic symptoms among subjects with ASD and the non-clinical population [41].

The psychological feature known as alexithymia pertains to the inability of an individual to identify and categorize their own emotions [50]. Somatic symptoms and physical health are believed to be closely related to alexithymia through a variety of mechanisms [51]. For instance, people with high levels of alexithymia may find it difficult to identify internal symptoms, and on the other hand, the physical feelings that come with emotional reactions may be mistaken for physical symptoms [36,41,52]. Indeed, research has shown that patients presenting with chronic pain have higher levels of alexithymia compared to control groups and that alexithymia is strongly associated with an over-reporting of physical symptoms [53,54]. Therefore, it stands to reason that people who have alexithymia may interpret and react to internal body cues in unusual ways [55]. Interestingly, about 50% of autistic subjects show elevated levels of alexithymia, while the majority have at least some alexithymic features [56,57,58]. This characteristic may predispose autistic people to experience and react to bodily sensations in altered ways, thus providing an explanation for the high prevalence of SDD in this population [59].

Another characteristic reported in SSD, which autistic people exhibit more than the general population [60,61], is intolerance of uncertainty [41]. Intolerance of uncertainty is the propensity to see uncertainty as dangerous and is associated with an increased risk of developing a number of anxiety disorders [62,63]. Moreover, by affecting how physical symptoms are interpreted, intolerance of uncertainty may unintentionally contribute to the development and maintenance of SSD [64].

Lastly, it has been highlighted how problems in social interaction can cause anxiety and stress, which might result in physical symptoms through a process known as “somatization” [14,65]. Indeed, recent studies have confirmed higher levels of somatization in ASD subjects compared to the non-clinical population [39,66,67], suggesting that some symptomatology experienced by autistic subjects is likely to stem from behavioral and emotional factors [68].

This review should be considered in light of some limitations. In particular, its nature as a narrative state-of-the-art review and therefore as a “transversal” method of investigation exposes it to the possibility of distorting the general picture of the development of a sector. However, this kind of review allows the reader to get an idea of the quantity and main characteristics of a topic without having to read multiple articles describing specific developments.

In conclusion, our review has collected the evidence available on how subjects with ASD report higher rates of SSD compared to the general population and how patients suffering from SSD have higher rates of autistic features. This is in line with studies that indicate that individuals with autism have higher rates of mental and physical disorders [69,70]. The data collected also support the theory that individuals with ASD would have higher somatic symptom levels, indicating that there may be a greater correlation between autism and physical discomfort [41]. Furthermore, regardless of the underlying medical condition, somatic symptoms are linked to increased functional impairment and suffering [71,72]. Given that autistic people are more susceptible to psychosocial stresses [73], stress and social situations are likely to have an effect on their physical health [74]. This may help to explain these increased physical symptoms. Noticeably, the link between SSD and ASD may add to the increasing research about female presentations of autism spectrum disorder, considering that SSD is known to be more frequent among females and a frequent comorbidity with other conditions, such as BPD and FED, which are reported to be associated with female ASD presentations [18,19,20,21,22,24,26,75].

Despite some promising results, to date, the available literature is still scant and limited to specific populations. In view of this, future studies should concentrate on additional factors that could account for the co-occurrence of ASD and SSD. An enhanced comprehension of plausible mechanisms and variables that could account for the simultaneous occurrence of somatic symptoms and autistic-like traits would be beneficial in enhancing preventive and therapeutic approaches for co-occurring disorders.

## Figures and Tables

**Table 1:** Autistic traits in SSRDs—summary table.

Reference	Sample	Methods	Results
Hatta et al. (2019) [27]	HC: N = 26 (F = 12; M = 14; mean age: 12.7 ± 1.9); SSD: N = 28 (F = 15; M = 13; mean age: 13.4 ± 2.0)	AQ—children’s version	42.9% of individuals with SSD had an AQ score above the average Higher scores in the AQ “Attention Switching” domain were associated with reduced health-related quality of life
Nisticò et al. (2022) [31]	NA: N = 45 (F = 17; M = 28; mean age: 35.36 ± 11.85); ASD: N = 30 (F = 14; M = 16; mean age: 39.67 ± 12.18) FNDs: N = 21 (F = 17; M = 4; mean age: 42.9 ± 13.02)	RAADS-R; AQ; FNS; SPQ-SF35.	86.7% of individuals with ASD reported at least one FNS vs. 35.6% of NAs Individuals with FNDs did not exhibit more autistic traits than NAs
Cole et al. (2023) [36]	FND: N = 91 (F = 69; M = 22; mean age: 43.42 ± 13.39)	AQ-10; PHQ-15; PHQ9; ASRS; TASS-20; GAD-7; WSAS.	36 subjects scored above the AQ-10 threshold 36 subjects screened positive for alexithymia AQ-10 had a direct effect on somatic symptoms Significant association between autistic traits and PHQ9 depression scores mediated by alexithymia score
Miyawaki et al. (2016) [37]	10-year-old girl with undiagnosed ASD and PNES	Pervasive Developmental Disorders Autism Society Japan Rating Scale; Children’s Global Assessment Scale	PNES as an outcome of psychological distress
McWilliams et al. (2019) [38]	ASD: N = 10 (F = 6; M = 4) HC: N = 49 (F = 31; M = 18)	Video EEG	10 subjects with non-epileptic seizures had clinically diagnosed ASD, and 5 had undiagnosed ASD

**Table 2:** SSRDs in ASD—summary table.

Reference	Sample	Methods	Results
Asztely et al. (2019) [39]	N = 77 (F = 77; mean age: 27.2 ± 4.2)	SF-36	76% of the subjects reported chronic pain and a low quality of life
Zdankiewicz-Cigaa et al. (2021) [40]	ASD: N = 79 (F = 57; M = 22; mean age: 35.12 ± 6.32) HC: N = 126 (F = 99; M = 27; mean age: 34.77 ± 9.54)	TAS-20; AQ; BPQ-SF; DERS; SDQ-20	People with ASD scored higher on the AQ TAS-20, BPQ-SF autonomic reactivity subscale, SDQ-20, and DERS
Larkin et al. (2023) [41]	N = 202 (F = 146; M = 48; other = 8; mean age = 31.33 ± 12.90) ASD: N = 51 (F = 29; M = 17; mean age: 33.9 ± 13.5) self-suspected ASD: N = 32 (F = 19; M = 10; mean age: 36.1 ± 13.5) without ASD: N = 119 (F = 98; M = 21; mean age: 28.9 ± 12.0)	PHQ15; AQ-10; TAS-20; BAQ; IUS-12	Subjects with ASD showed more somatic symptoms than the non-ASD group; the self-suspected ASD group showed intermediate levels of symptoms; and the self-suspected ASD group showed significantly lower levels of interoception than the ASD group
Williams et al. (2019) [42]	ASD: N = 290 (F = 177; M = 113; mean age: 23.10 ± 2.38)	PHQ15; SRS-II; GAD-7; BDI-II; WHOQOL-4	Significantly higher levels of somatic symptoms in the ASD group, with most common symptoms being exhaustion, sleep issues, and menstruation issues

## Data Availability

No new data were created or analyzed in this study. Data sharing is not applicable to this article.

## References

[1] American Psychiatric Association (2022). Diagnostic and Statistical Manual of Mental Disorders.

[2] Afif I.Y., Manik A.R., Munthe K., Maula M.I., Ammarullah M.I., Jamari J., Winarni T.I. (2022). Physiological Effect of Deep Pressure in Reducing Anxiety of Children with ASD during Traveling: A Public Transportation Setting. Bioengineering.

[3] Kinnaird E., Stewart C., Tchanturia K. (2019). Investigating alexithymia in autism: A systematic review and meta-analysis. Eur. Psychiatry.

[4] Poquérusse J., Pastore L., Dellantonio S., Esposito G. (2018). Alexithymia and Autism Spectrum Disorder: A complex relationship. Front. Psychol..

[5] Dell’Osso L., Lorenzi P., Carpita B. (2019). The neurodevelopmental continuum towards a neurodevelopmental gradient hypothesis. J. Psychopathol..

[6] Dell’Osso L., Lorenzi P., Carpita B. (2019). Autistic Traits and Illness Trajectories. Clin. Pract. Epidemiol. Ment. Health.

[7] Baron-Cohen S., Leslie A.M., Frith U. (1985). Does the autistic child have a “theory of mind”?. Cognition.

[8] Rosen N.E., Lord C., Volkmar F.R. (2021). The diagnosis of autism: From kanner to DSM-III to DSM-5 and beyond. J. Autism Dev. Disord..

[9] White S.W., Oswald D., Ollendick T., Scahill L. (2009). Anxiety in children and adolescents with autism spectrum disorders. Clin. Psychol. Rev..

[10] Cerutti R., Presaghi F., Spensieri V., Valastro C., Guidetti V. (2016). The potential impact of internet and mobile use on headache and other somatic symptoms in adolescence. A populationbased cross-sectional study. Headache.

[11] Cerutti R., Spensieri V., Valastro C., Presaghi F., Canitano R., Guidetti V. (2017). A comprehensive approach to understand somatic symptoms and their impact on emotional and psychosocial functioning in children. PLoS ONE.

[12] Korterink J.J., Diederen K., Benninga M.A., Tabbers M.M. (2015). Epidemiology of pediatric functional abdominal pain disorders: A meta-analysis. PLoS ONE.

[13] Chang L. (2011). The role of stress on physiologic responses and clinical symptoms in irritable bowel syndrome. Gastroenterology.

[14] Beck J.E. (2008). A developmental perspective on functional somatic symptoms. J. Pediatr. Psychol..

[15] Natalucci G., Faedda N., Calderoni D., Cerutti R., Verdecchia P., Guidetti V. (2018). Headache and alexithymia in children and adolescents: What is the connection?. Front. Psychol..

[16] Sifneos P.E. (1973). The prevalence of ‘alexithymic’ characteristics in psychosomatic patients. Psychother. Psychosom..

[17] Corbett B.A., Schupp C.W., Levine S., Mendoza S. (2009). Comparing cortisol, stress, and sensory sensitivity in children with autism. Autism Res..

[18] Dell’Osso L., Carpita B. (2023). What misdiagnoses do women with autism spectrum disorder receive in the DSM-5?. CNS Spectr..

[19] Carpita B., Muti D., Cremone I.M., Fagiolini A., Dell’Osso L. (2022). Eating disorders and autism spectrum: Links and risks. CNS Spectr..

[20] Dell’Osso L., Abelli M., Pini S., Carpita B., Carlini M., Mengali F., Tognetti R., Rivetti F., Massimetti G. (2015). The influence of gender on social anxiety spectrum symptoms in a sample of university students. Riv. Psichiatr..

[21] Marazziti D., Abelli M., Baroni S., Carpita B., Piccinni A., Dell’Osso L. (2014). Recent findings on the pathophysiology of social anxiety disorder. Clin. Neuropsychiatry.

[22] Dell’Osso L., Lorenzi P., Carpita B. (2021). Camouflaging: Psychopathological meanings and clinical relevance in autism spectrum conditions. CNS Spectr..

[23] Cremone I.M., Carpita B., Nardi B., Casagrande D., Stagnari R., Amatori G., Dell’Osso L. (2023). Measuring Social Camouflaging in Individuals with High Functioning Autism: A Literature Review. Brain Sci..

[24] Dell’Osso L., Cremone I.M., Amatori G., Cappelli A., Cuomo A., Barlati S., Massimetti G., Vita A., Fagiolini A., Carmassi C. (2021). Investigating the Relationship between Autistic Traits, Ruminative Thinking, and Suicidality in a Clinical Sample of Subjects with Bipolar Disorder and Borderline Personality Disorder. Brain Sci..

[25] Dell’Osso L., Cremone I.M., Nardi B., Tognini V., Castellani L., Perrone P., Amatori G., Carpita B. (2023). Comorbidity and Overlaps between Autism Spectrum and Borderline Personality Disorder: State of the Art. Brain Sci..

[26] Carpita B., Nardi B., Pronestì C., Parri F., Giovannoni F., Cremone I.M., Pini S., Dell’Osso L. (2023). May Female Autism Spectrum Be Masked by Eating Disorders, Borderline Personality Disorder, or Complex PTSD Symptoms? A Case Series. Brain Sci..

[27] Hatta K., Hosozawa M., Tanaka K., Shimizu T. (2019). Exploring Traits of Autism and Their Impact on Functional Disability in Children with Somatic Symptom Disorder. J. Autism Dev. Disord..

[28] Kimura M., Chisaki K., Yamaguchi H., Ikuno T. (2006). Underlying developmental disorders in patients who visited the outoatient psychosomatic clinic of the development of pediatrics. Jpn. J. Psychosom. Res..

[29] Nitahara Y., Tachibana Y., Koeda T., Okuyama M. (2015). Kirituseicyousetusyougai no haikei ni jiheisyousupekutoramu wo mitometa 1 rei. Nippon. Rinsho.

[30] Shiokawa H., Momoi M. (2002). Psychosomatic and behavioral problems in children with Asperger disorder. Jpn. J. Psychosom. Med..

[31] Nisticò V., Goeta D., Iacono A., Tedesco R., Giordano B., Faggioli R., Priori A., Gambini O., Demartini B. (2022). Clinical overlap between functional neurological disorders and autism spectrum disorders: A preliminary study. Neurol. Sci..

[32] Duddu V., Isaac M.K., Chaturvedi S.K. (2003). Alexithymia in somatoform and depressive disorders. J. Psychosom. Res..

[33] Tominaga T., Choi H., Nagoshi Y., Wada Y., Fukui K. (2014). Relationship between alexithymia and coping strategies in patients with somatoform disorder. Neuropsychiatr. Dis. Treat..

[34] Ak I., Sayar K., Yontem T. (2004). Alexithymia, somatosensory amplification and counter-dependency in patients with chronic pain. Pain Clin..

[35] Heniquez A., Lahaye H., Boissel L., Guilé J.M., Benarous X. (2022). Spécificités intéroceptives chez les enfants et adolescents qui présentent un trouble à symptomatologie somatique: Étude descriptive sur dix-neuf patients [Interoceptive difficulties in children and adolescents with severe form of somatic symptom disorder: A pilot study with nineteen participants]. Encephale.

[36] Cole R.H., Elmalem M.S., Petrochilos P. (2023). Prevalence of autistic traits in functional neurological disorder and relationship to alexithymia and psychiatric comorbidity. J. Neurol. Sci..

[37] Miyawaki D., Iwakura Y., Seto T., Kusaka H., Goto A., Okada Y., Asada N., Yanagihara E., Inoue K. (2016). Psychogenic nonepileptic seizures as a manifestation of psychological distress associated with undiagnosed autism spectrum disorder. Neuropsychiatr. Dis. Treat..

[38] McWilliams A., Reilly C., Gupta J., Hadji-Michael M., Srinivasan R., Heyman I. (2019). Autism spectrum disorder in children and young people with non-epileptic seizures. Seizure.

[39] Asztély K., Kopp S., Gillberg C., Waern M., Bergman S. (2019). Chronic Pain and Health-Related Quality of Life in Women with Autism and/or ADHD: A Prospective Longitudinal Study. J. Pain Res..

[40] Zdankiewicz-Ścigała E., Ścigała D., Sikora J., Kwaterniak W., Longobardi C. (2021). Relationship between interoceptive sensibility and somatoform disorders in adults with autism spectrum traits. The mediating role of alexithymia and emotional dysregulation. PLoS ONE.

[41] Larkin F., Ralston B., Dinsdale S.J., Kimura S., Hayiou-Thomas M.E. (2023). Alexithymia and intolerance of uncertainty predict somatic symptoms in autistic and non-autistic adults. Autism.

[42] Williams Z.J., Gotham K.O. (2022). Current and lifetime somatic symptom burden among transition-aged autistic young adults. Autism Res..

[43] Aldinger K.A., Lane C.J., Veenstra-VanderWeele J., Levitt P. (2015). Patterns of risk for multiple co-occurring medical conditions replicate across distinct cohorts of children with autism spectrum disorder. Autism Res..

[44] Fulceri F., Morelli M., Santocchi E., Cena H., Del Bianco T., Narzisi A., Calderoni S., Muratori F. (2016). Gastrointestinal symptoms and behavioral problems in preschoolers with Autism Spectrum Disorder. Dig. Liver Dis..

[45] Kuhlthau K.A., McDonnell E., Coury D.L., Payakachat N., Macklin E. (2018). Associations of quality of life with health-related characteristics among children with autism. Autism.

[46] Hogendoorn E., Hartman C.A., Burke S.M., van Dijk M.W.G., Rosmalen J.G.M. (2023). Longitudinal relations between autistic-like features and functional somatic symptoms in adolescence. Autism.

[47] Garfinkel S.N., Tiley C., O’Keeffe S., Harrison N.A., Seth A.K., Critchley H.D. (2016). Discrepancies between dimensions of interoception in autism: Implications for emotion and anxiety. Biol. Psychol..

[48] Jenkinson R., Milne E., Thompson A. (2020). The relationship between intolerance of uncertainty and anxiety in autism: A systematic literature review and meta-analysis. Autism.

[49] Vasa R.A., Kreiser N.L., Keefer A., Singh V., Mostofsky S.H. (2018). Relationships between autism spectrum disorder and intolerance of uncertainty. Autism Res..

[50] Kafetsios K., Hess U. (2019). Seeing mixed emotions: Alexithymia, emotion perception bias, and quality in dyadic interactions. Pers. Individ. Differ..

[51] Porcelli P., Taylor G., Luminet O., Bagby R., Taylor G. (2018). Alexithymia and physical illness: A psychosomatic approach. Alexithymia: Advances in Research, Theory, and Clinical.

[52] Kojima M. (2012). Alexithymia as a prognostic risk factor for health problems: A brief review of epidemiological studies. Biopsychosoc. Med..

[53] Kreitler S., Niv D. (2001). Pain and alexithymia: The nature of a relation. Pain Clin..

[54] Lumley M.A., Neely L.C., Burger A.J. (2007). The assessment of alexithymia in medical settings: Implications for understanding and treating health problems. J. Pers. Assess..

[55] Kano M., Fukudo S. (2013). The alexithymic brain: The neural pathways linking alexithymia to physical disorders. Biopsychosoc. Med..

[56] Bird G., Viding E. (2014). The self to other model of empathy: Providing a new framework for understanding empathy impairments in psychopathy, autism, and alexithymia. Neurosci. Biobehav. Rev..

[57] Hill E., Berthoz S., Frith U. (2004). Brief report: Cognitive processing of own emotions in individuals with autistic spectrum disorder and in their relatives. J. Autism Dev. Disord..

[58] Lombardo M.V., Barnes J.L., Wheelwright S.J., Baron-Cohen S. (2007). Self-referential cognition and empathy in autism. PLoS ONE.

[59] Williams Z.J., Gotham K.O. (2021). Improving the measurement of alexithymia in autistic adults: A psychometric investigation of the 20-item Toronto Alexithymia Scale and generation of a general alexithymia factor score using item response theory. Mol. Autism.

[60] Boulter C., Freeston M., South M., Rodgers J. (2014). Intolerance of uncertainty as a framework for understanding anxiety in children and adolescents with autism spectrum disorders. J. Autism Dev. Disord..

[61] Neil L., Olsson N.C., Pellicano E. (2016). The relationship between intolerance of uncertainty, sensory sensitivities, and anxiety in autistic and typically developing children. J. Autism Dev. Disord..

[62] Birrell J., Meares K., Wilkinson A., Freeston M. (2011). Toward a definition of intolerance of uncertainty: A review of factor analytical studies of the Intolerance of Uncertainty Scale. Clin. Psychol. Rev..

[63] Rodgers J., Herrema R., Honey E., Freeston M. (2018). Towards a Treatment for Intolerance of Uncertainty for Autistic Adults: A Single Case Experimental Design Study. J. Autism Dev. Disord..

[64] Carleton R.N. (2012). The intolerance of uncertainty construct in the context of anxiety disorders: Theoretical and practical perspectives. Expert. Rev. Neurother..

[65] Spain D., Sin J., Linder K.B., McMahon J., Happé F. (2018). Social anxiety in autism spectrum disorder: A systematic review. Res. Autism Spectr. Disord..

[66] Lever A.G., Geurts H.M. (2016). Psychiatric co-occurring symptoms and disorders in young, middle-aged, and older adults with autism spectrum disorder. J. Autism Dev. Disord..

[67] Okamoto Y., Yoshihara M., Miyake Y., Nagasawa I. (2017). Young adults with autism spectrum disorder: Intervention to psychosomatic symptoms of childhood is the key preventing maladjustment of the youth. Acta Psychopathol..

[68] Pan P.Y., Tammimies K., Bölte S. (2020). The Association Between Somatic Health, Autism Spectrum Disorder, and Autistic Traits. Behav. Genet..

[69] Lai M.C., Kassee C., Besney R., Bonato S., Hull L., Mandy W., Szatmari P., Ameis S.H. (2019). Prevalence of co-occurring mental health diagnoses in the autism population: A systematic review and meta-analysis. Lancet Psychiatry.

[70] Mason D., Ingham B., Urbanowicz A., Michael C., Birtles H., Woodbury-Smith M., Brown T., James I., Scarlett C., Nicolaidis C. (2019). A systematic review of what barriers and facilitators prevent and enable physical healthcare services access for autistic adults. J. Autism Dev. Disord..

[71] Sharpe M. (2013). Somatic symptoms: Beyond ‘medically unexplained’. Br. J. Psychiatry.

[72] Trimble M. (2004). Somatoform Disorders: A Medicolegal Guide.

[73] Griffiths S., Allison C., Kenny R., Holt R., Smith P., Baron-Cohen S. (2019). The Vulnerability Experiences Quotient (VEQ): A Study of Vulnerability, Mental Health and Life Satisfaction in Autistic Adults. Autism Res..

[74] Kirmayer L.J., Groleau D., Looper K.J., Dao M.D. (2004). Explaining medically unexplained symptoms. Can. J. Psychiatry.

[75] Barthels F., Müller R., Schüth T., Friederich H.C., Pietrowsky R. (2021). Orthorexic eating behavior in patients with somatoform disorders. Eat. Weight Disord..

